# Evaluation of (Shared) Autonomy in Robot‐Assisted Vitreoretinal Surgery Using a Surgical Model

**DOI:** 10.1002/rcs.70040

**Published:** 2025-01-09

**Authors:** Murilo M. Marinho, Yuki Koyama, Yuta Taniguchi, Toshiro Yamanaka, Fumihito Arai, Seiji Omata, Koichiro Sugimoto, Takashi Ueta, Kiyohito Totsuka, Tomoyasu Shiraya, Fumiyuki Araki, Muneyuki Takao, Mamoru Mitsuishi, Kanako Harada

**Affiliations:** ^1^ Department of Mechanical Engineering The University of Tokyo Bunkyo Japan; ^2^ Department of Electrical and Electronic Engineering The University of Manchester Manchester UK; ^3^ Faculty of Advanced Science of Technology Kumamoto University Kumamoto Japan; ^4^ Department of Ophthalmic Surgery The University of Tokyo Hospital Bunkyo Japan; ^5^ Advanced Comprehensive Research Organization, Teikyo University Itabashi Japan

**Keywords:** bimanual, kinematics, teleoperation

## Abstract

**Background:**

Robot‐assisted vitreoretinal surgery makes it easier for the surgeons to perform precise and dexterous manipulations required in vitreoretinal procedures.

**Methods:**

We systematically evaluated manual surgery, conventional two‐hand teleoperation, a novel one‐hand teleoperation, and automation in a needle positioning task using a realistic surgical eye model, measuring the expert surgeon's performances and the novice's learning curves.

**Results:**

The proposed one‐hand teleoperation improved the positioning accuracy of expert surgeons (p<0.05), enabled novices to achieve a consistent accuracy more quickly (p<0.05), decreased the novice's workload more quickly (p<0.05), and made it easier for novices to learn to conduct the task quickly (p<0.05). Moreover, our autonomous positioning achieved an equivalent accuracy to the surgeons.

**Conclusions:**

The benefits and potential of task autonomy were shown. Further work is needed to evaluate the proposed methods in a more complex task.

## 1 Introduction

Ophthalmic surgical procedures that can benefit from robotic assistance include the peeling of 2.5 μm thick inner limiting membrane and the cannulation of retinal blood vessels with less than 100 μm in diameter. These difficulties are well known because the average amplitude of hand tremor is approximately 100μm [1], and the low force requirements of (usually) less than 7.5mN [2].

In this context, much interest in the community has been directed towards the development of ophthalmic‐specific robotic systems [3]. For instance, Wilson et al. [4] developed the Intraocular Robotic Interventional Surgical System (IRISS) and their integrated system could perform various vitreoretinal surgical tasks [5, 6]. A robotic system capable of both extraocular and intraocular manipulation was presented by Yu et al. [7, 8]. Nasseri et al. [9] introduced a compact robot that adopted a hybrid parallel‐serial mechanism. They integrated this robot with intraoperative optical coherence tomography and proposed a system for sub‐retinal injection [10]. Micron was developed by MacLachlan et al. [11], which has the benefits of tremor suppression and intuitive operation. In addition, they have been working on the new design of Micron [12, 13]. The Steady‐Hand Eye Robot (SHER) of Johns Hopkins University [14, 15] has been the root of many other pieces of research. For instance, He et al. [16] investigated the force‐sensing forceps with Fibre Bragg Gratings optical sensors, and Patel et al. [17] succeeded to maintain scleral forces within safe limits using the SHER incorporated with the force‐sensing forceps. Gijbels et al. [18] have achieved first‐in‐human retinal vein cannulation, where the robot succeeded in maintaining the microneedle's position throughout the 10‐min‐injection. Only the Preceyes Surgical Robotic System (Preceyes B.V., the Netherlands) has a CE mark for everyday use in ocular surgery and was used in the successful first human intraocular robotic surgery [19].

Using these ophthalmic‐specific platforms, Maberley et al. [20] compared the robot‐assisted (with the Preceyes Surgical Robotic System) surgery with manual surgery in a peeling task. Their results show that retinal injuries were eliminated, although the average time increased. Jacobsen et al. [21] evaluated learning curves of the subjects using a modified Preceyes Surgical Robotic System. They concluded that there was no significant time difference for the learning curve to plateau, while robot‐assisted vitreoretinal surgery was associated with less instrument movement and less tissue damage. In their results, the challenges of the robot‐assisted surgery are still pointed out, and significant differences have not yet been obtained. These results are in tune with the results obtained with teleoperated robots in other specialities, in the sense that the increased precision allowed by the robotic assistance is often associated with a longer operative time.

(Semi‐)automation is believed to be one way to obtain high precision without a large increase in operating times. Virtual fixtures, a semi‐automation strategy based altering robot motion based on virtual regions, and force feedback has been explored. For instance, Nasseri et al. [22] investigated virtual fixtures to protect specific regions of the eye. Yang et al. [23] have used virtual fixtures and force feedback to generate spherical and cylindrical volumes in the eye in the context of cataract surgery. To the best of our knowledge, comprehensive studies comparing (semi‐)automation with manual operation are still not available.

With respect to automation, Shin et al. [6] used the IRISS coupled with OCT as visual feedback to the robotic system for partial automation [In their work they refer to semi‐automation, but in the terminology of this work we can call it partial automation given that part of the task did not have human involvement]. They used deep learning for semantic scene understanding for semi‐automated extraction of lens fragments. Given the difficulty of the procedure, only part of it was automated, but future work aims to achieve full automation. Towards the automation of another subtask, Dehghani et al. [24] investigated the automation of the trocar docking and instrument insertion into the eye using a 5 DoF hybrid parallel‐serial robot. This study is one important step towards the full automation of eye surgical procedures.

Using a general‐purpose platform for surgical robotics research, called SmartArm [25], we have explored teleoperation, shared autonomy, and automation in applications related to several surgical specialities. Among them, we have recently investigated its use in ophthalmic surgery automation, aiming applications such as retinal vein cannulation [26] and peeling [27]. In this work, we are interested in systematically evaluating manual surgery, conventional two‐hand teleoperation, a novel one‐hand teleoperation, and automation using a realistic surgical eye model.

### 1.1 Statement of Contributions

The main contributions of this work are:

1. An introduction of a novel one‐hand teleoperative control system that sets one hand of the surgeon free;
2. Experiments comparing manual surgery, robot surgery (regular two‐hand teleoperation), and robot surgery with assistance (novel one‐hand teleoperation) in a needle positioning task using a realistic surgical eye model [28] in two settings
  - task performance and workload for expert surgeons;
  - learning curves of novices.
3. An experiment that evaluated autonomous positioning (full‐autonomy) using the same positioning task.

## 2 Methods

This section first explains our system architecture and its control strategies under three different levels of autonomy: telae‐operation, shared‐autonomy, and full‐autonomy. From Section 2.3, we describe the design of two user studies and one experiment we conducted to evaluate our control strategies. The results and discussion of the user studies and the experiment are described in Section 3 and 4, respectively.

In the user experiments, all subjects gave written consent after receiving an explanation regarding the experiment before the trials began. The experimental protocol was approved by the Research Ethics Committee of the Faculty of Medicine of the University of Tokyo (Protocol number: 2022174NI, Date of Approval: 3rd October 2022).

### 2.1 SmartArm Surgical Robotic System

**FIGURE 1:**
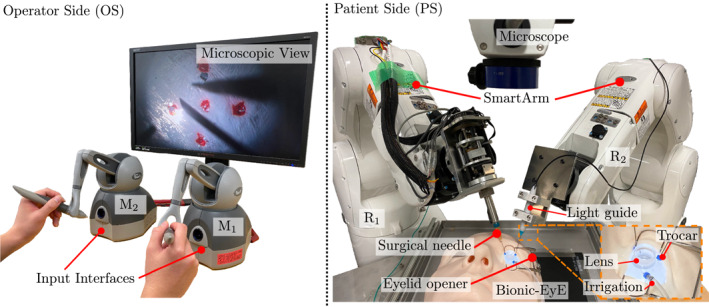
The vitreoretinal setup of our SmartArm surgical robotic system [25]. The left side shows an operator side, where a surgeon operates input interfaces, $M_{1}$ and $M_{2}$. The right side is a patient side, where the robot $R_{1}$ holds a surgical needle, and the robot $R_{2}$ has a light guide. The vitreoretinal surgical phantom called the Bionic‐EyE [29] is place between the two robots.

Consider the SmartArm surgical robotic system outfitted for vitreoretinal surgery shown in Figure 1, composed of an operator side and a patient side.

The operator side consists of two input interfaces (Touch, 3D Systems, USA). The interfaces $M_{i}\;\left(i=1,\;2\right)$ are handled by the operator's right hand and left hand, respectively. In our setup, $M_{1}$ controls the tip position of the dominant instrument, and $M_{2}$ controls the tip position of the light guide. The operator sees the 2D microscopic images on a monitor.

The patient side consists of two robotic arms $R_{i}$ (VS050, DensoWave, Japan) with six degrees‐of‐freedom, hence each has a configuration space $q_{i}\in R^{6}\;\left(i=1,\;2\right)$. The robot $R_{1}$ holds a surgical needle [Other instruments like surgical forceps are also used depending on the task.] as a dominant surgical instrument, and the robot $R_{2}$ holds a light guide. Each input interface is used to control its respective robot $R_{i}$. The system runs on an updated version of the SmartArm system software [25]. Both instruments are inserted into an eye model of the vitreoretinal surgical simulator called the Bionic‐EyE [29] placed between the two robots. Moreover, a disposable flat lens is put to provide the correct view of the workspace. An ophthalmic microscope is placed above the Bionic‐EyE.

In the evaluations, we use the four following operative modes:

1. Manual operation (**MO**): the operator performs the procedure manually, without using the robots.
2. Teleoperation (**TO**): the operator controls both instruments using the input interfaces $M_{1}$ and $M_{2}$.
3. Shared autonomy (**SA**): the operator controls only the dominant instrument using the input interface $M_{1}$. At the same time, the system autonomously controls the light guide.
4. Full autonomy (**FA**): the two robotic arms perform the task autonomously based on the information obtained using image processing.

### 2.2 Control Strategies

This section describes the details of the three robotic control strategies, **TO**, **SA**, and **FA**. All strategies rely on the teleoperation framework in unit‐dual‐quaternion space [30], that uses the vector‐field‐inequalities (VFIs) [31] for the generation of virtual fixtures. In addition, we use the coordinated control of the light guide proposed in our previous work [26] to autonomously control the light guide in **SA** and **FA**.

#### 2.2.1 Patient Side Control

In the patient side, the translations [We control only the translations of the instruments' tips because of their symmetry with respect to their shafts.] of the instruments' tips are controlled by a centralised kinematic control strategy [30], using a quadratic programme as follows

(1) $$\begin{array}{ll} u\in \underset{\dot{q}}{\mathrm{arg min}} & \beta F_{1}\left(q_{1},\;t_{1,d}\right)+\left(1-\beta \right)F_{2}\left(q_{2},\;t_{2,d}\right)\\ \text{subject}\;\text{to} & W\dot{q}⪯w, \end{array}$$

where $q={\left[\begin{array}{cc} q_{1}^{T} & q_{2}^{T} \end{array}\right]}^{T}$ is the joint configuration of the entire system, $F_{i}\left(q_{i},\;t_{i,d}\right)\;\left(i=1,\;2\right)$ are quadratic objective functions related to the i‐th robot. In **TO** and **SA**, the desired translation $t_{1,d}≜t_{1,d}\left(t\right)$ is, mutatis mutandis, sent from $M_{1}$. In **FA**, image processing is used to get $t_{1,d}$. The desired translation $t_{2,d}≜t_{2,d}\left(t\right)$ is sent from $M_{2}$ in **TO,** whereas $t_{2,d}$ is set to the current position in **SA** and **FA**, which means that the light guide will not move from the current position if not necessary. The parameter β∈[0,1]⊂R is a weight that defines a priority between the two robots. This value defines which robot to prioritise when both objective functions cannot be individually fulfiled without the violation of the constraints. To let the system prefer $t_{1,d}$ convergence over $t_{2,d}$ convergence in such a situation, β was set to 0.99 in all setups [Please note that we can not choose the value of β=1 since this leads the light guide behaving unpredictably.]. The r inequality constraints constructed with $W≜W\left(q\right)\in R^{r\times 12}$, $w≜w\left(q\right)\in R^{r}$ are used to generate active constraints using the VFI methodology [31]. The optimal control signal u is sent to the robots at each time step.

The inequality constraints $W\dot{q}⪯w$ are used to enforce safety and to control the light guide autonomously. The following sections explain the roles of these inequality constraints. Mathematical details of each constraint are explained in Section VIII and IX of our previous work [26]. The feasibility of the constraints and the impact on surgical safety have also been evaluated in Section X of our previous work [26].

#### 2.2.2 Inequality Constraints for Teleoperation (**TO**)

In **TO**, the following constraints are enforced for a safe operation using the VFI method:

- $C_{R}$: the shafts must always pass through their respective insertion points called the remote centre‐of‐motion (RCM),
- $C_{r}$: the light guide's tip must never touch the inner wall of the eye,
- $C_{s}$: the light guide's tip must never collide with the shaft of the surgical needle,
- $C_{\text{tr}}$: the instruments' tips must always remain inside the eye,
- $C_{j}$: the joint values must never exceed their limits.

**FIGURE 2:**
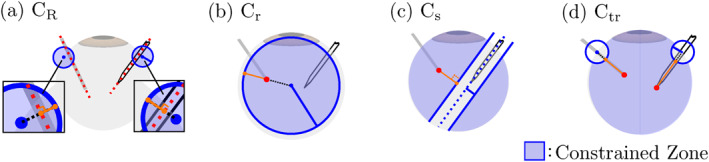
The geometric primitives used to enforce the constraints $C_{R}$, $C_{r}$, $C_{s}$, and $C_{\text{tr}}$. The red geometric primitives are constrained inside or outside the blue geometric primitives using the VFI method. The lengths of the singed distances, shown as orange lines, are kept positive.

The VFI method relies on a signed distance function between two geometric primitives, as shown in Figure 2. One geometric primitive is constrained inside or outside the other by keeping the value of the signed distance positive. The safety distances of each constraint were decided based on the permissible maximum motion error values evidenced in our previous work [32]. The inequality constraints for **TO** is described as follows

(2) $$\underset{W_{\text{TO}}}{\underbrace{\left[\begin{array}{c} W_{R}\\ W_{r}\\ W_{s}\\ W_{\text{tr}}\\ W_{j} \end{array}\right]}}\dot{q}⪯\underset{w_{\text{TO}}}{\underbrace{\left[\begin{array}{c} w_{R}\\ w_{r}\\ w_{s}\\ w_{\text{tr}}\\ w_{j} \end{array}\right]}}.$$

#### 2.2.3 Inequality Constraints for Shared Autonomy (**SA**)

The introduction of **SA** is one of the contributions of this work. In **SA**, the light guide is autonomously controlled with respect to the motion of the surgical needle controlled by the surgeon.

For this, in addition to the constraints (2), we use the two following constraints.

- $C_{t}$: the surgical needle's tip must be illuminated sufficiently, by keeping the light guide's tip within 8mm.
- $C_{2}$: the surgical needle's tip must be illuminated at all times, by keeping the tip inside the illumination cone of the light guide.

**FIGURE 3:**
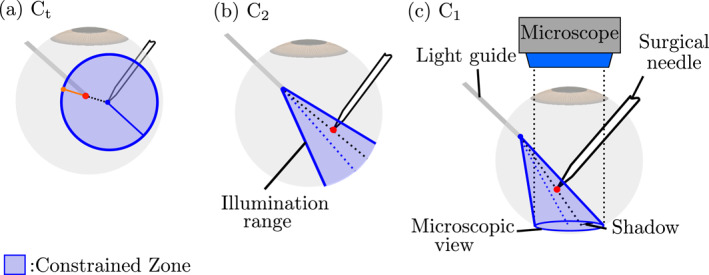
The geometric primitives used to enforce the constraints $C_{t}$, $C_{2}$, and $C_{1}$.

The geometrical primitives used to enforce $C_{t}$ and $C_{2}$ are shown in Figure 3. By adding these constraints to (2), the inequality constraints for **SA** is implemented as follows

(3) $$\underset{W_{\text{SA}}}{\underbrace{\left[\begin{array}{c} W_{\text{TO}}\\ W_{t}\\ W_{2} \end{array}\right]}}\dot{q}⪯\underset{w_{\text{SA}}}{\underbrace{\left[\begin{array}{c} w_{\text{TO}}\\ w_{t}\\ w_{2} \end{array}\right]}}.$$

In (1), the light guide will not move unless the constraints (3) are violated. That way, the light guide is autonomously controlled by the system in an optimal way.

#### 2.2.4 Inequality Constraints for Full Autonomy (**FA**)

In **FA**, to autonomously conduct tasks, the distance or proximity between the instrument's tip and the fundus must be estimated. To estimate the proximity, we proposed to use the shadow of the instrument projected on the fundus of the eye [26]. The distance between the instrument's tip and its shadow is related to the distance between the instrument's tip and the fundus. For this, the following constraint is added to (3):

- $C_{1}$: the tip of the instrument's shadow is always visible in the microscopic view.

The conical constraint $C_{1}$, shown in Figure 3, is used for this. The inequality constraints for **FA** is as follows

(4) $$\underset{W_{\text{FA}}}{\underbrace{\left[\begin{array}{c} W_{\text{SA}}\\ W_{1} \end{array}\right]}}\dot{q}⪯\underset{w_{\text{FA}}}{\underbrace{\left[\begin{array}{c} w_{\text{SA}}\\ w_{1} \end{array}\right]}}.$$

### 2.3 Design of User Experiments

**FIGURE 4:**
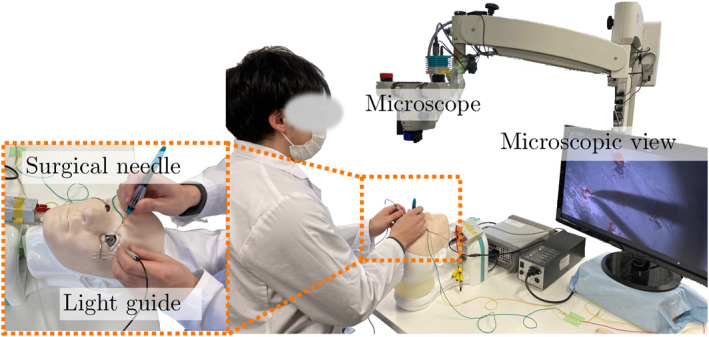
The manual setup compared to the robot‐assisted setups.

We conducted two user studies to compare **MO**, **TO**, and **SA**. The manual setup, **MO**, was prepared as shown in Figure 4. To ensure a fair comparison in terms of vision, the subjects were asked to perform the task using a monitor, instead of looking directly through the microscope. In the robotic setup, the surgeons cannot look into the microscope directly, since the patient side and the operator side are separated.

In the first user study, **U1**, detailed in Section 2.3.1, six expert surgeons were recruited. Our intention was to evaluate expert surgeon experience among different setups, while blocking for learning effects. In the second user study, **U2**, detailed in Section 2.3.2, 12 novice users were recruited to evaluate learning curves among different control strategies. Given the limited number of available participants, we recruited all subjects who met the inclusion criteria.

In both studies, the subjects were asked to perform a needle positioning task, and the metrics detailed in Section 2.3.4 were evaluated.

#### 2.3.1 User Study U1: Evaluation of Usability by Expert Surgeons

The first user study, **U1**, was conducted to evaluate **MO**, **TO**, and **SA** among expert surgeons. For this, six ophthalmic expert surgeons with more than 10 years of vitreoretinal surgery experience from the University of Tokyo Hospital, Japan, were recruited. Each surgeon was asked to complete the positioning task five times for each one of the three setups.

To reduce learning effects related to the differences between real surgery and our experimental setups, all subjects performed 2 min of practice. Moreover, to block for learning effects in the robot‐assisted setups, one half of the subjects started from the **TO** setup, and the other half started from the **SA** setup. Owing to a considerable time requirement in switching between robot‐assisted and manual setups, all subjects performed the **MO** trials last.

#### 2.3.2 User Study U2: Evaluation of Novice's Learning Curve

In the second user study, **U2**, the learning curves of novices under the three setups were evaluated: **MO**, **TO**, and **SA**. To get the learning curves, 12 novice volunteers from the graduate students of the Schools of Engineering of the University of Tokyo were recruited.

The 12 novices were equally divided into the three setups and asked to conduct the positioning task 10 times using only the setup they were assigned to. No practice was performed before this evaluation.

##### 2.3.2.1 Analysis of the Learning Curves

The learning curves are depicted based on the median values of the four subjects. Using the log‐linear model [33] with an additive constant $\left(Y=B+Cx^{-b}\right)$, a decreasing curve was fit for the median values. The coefficients, B,C,b, were determined so that the learning curve passed through the median value of the first trial $m_{1}$ $\left(m_{1}=B+C\right)$, and the sum of squared distances from the other median values was minimised.

To compare the learning curves among the setups, learning rate, learning plateau, and the area under learning curve (AULC) were used. In log‐linear model, $Y=B+Cx^{-b}$, the exponent part b and the additive constant B were defined as the learning rate and the learning plateau, respectively. The learning plateau is the asymptote of the learning curve, which represents the theoretical best value a subject can achieve with infinite practice. A small value of AULC means that the task is easy for the subject to learn.

#### 2.3.3 Positioning Task

**FIGURE 5:**
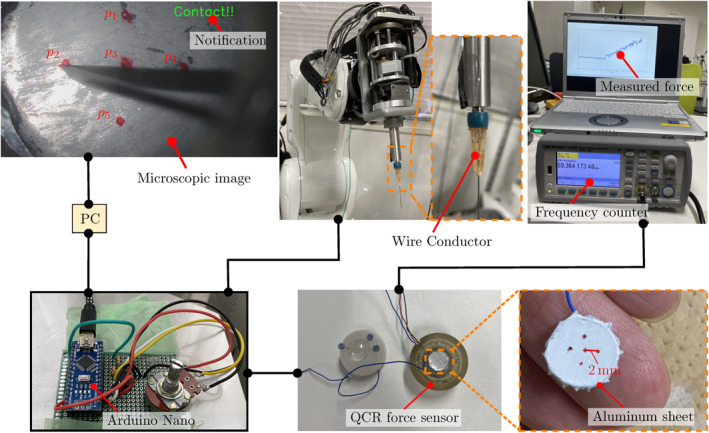
The system for contact and force measurement. The contact between the surgical needle and the aluminium sheet closes a circuit in such way that it can be detected by a microcontroller. The force applied to the fundus of the eye model was measured through a quartz crystal resonator force sensor [28].

The positioning task is part of a more complex operation but the step with the highest risk of damaging the retina. Therefore, we first focused on this step to reveal the impact of the proposed system. In the studies, the task was conducted inside the workspace of the fundus of the eye model. As shown in Figure 5, five red target points, $p\in \left\{p_{1},\;p_{2},\;p_{3},\;p_{4},\;p_{5}\right\}$, were drawn, by hand, on the fundus. The point $p_{3}$ was located at the centre of the workspace [In our group, the workspace was defined as a 7mm diameter circular region in the retina after discussions with partner expert surgeons [32].]. The other points were located at the 4mm diameter circle centred at $p_{3}$, to match with the uniform force sensitivity region of the force sensor [28].

The users were asked to position the surgical needle's tip to the five target points in crescent order, as quickly and accurately as possible.

A common criterion for task completion is needed to reduce inter‐participant variability. For this, contact with each target was relayed to the user using the system shown in Figure 5. The system can detect the contact between the tip and the fundus using electric current. When the electric conduction is detected, the word ‘Contact!!’ was shown in the monitor.

#### 2.3.4 Evaluation Metrics

The evaluated metrics were positioning accuracy, the operator's workload calculated using the NASA‐TLX [34], the force applied on the fundus, and task duration.

##### 2.3.4.1 Positioning Accuracy

**FIGURE 6:**
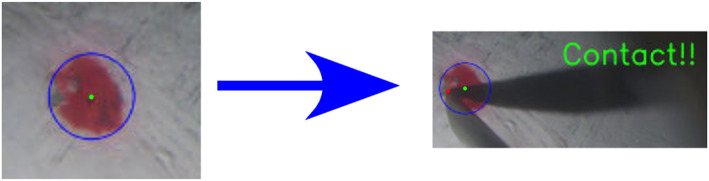
Definition of the positioning accuracy.

To evaluate accuracy, the positioning error was calculated from recorded Video 1. Firstly, a circle that includes the red target point was set as shown in Figure 6. Then, the distance between the centre and the positioning point was defined as accuracy.

**VIDEO 1:** To view this video in the full‐text HTML version of the article, please visit https://onlinelibrary.wiley.com/doi/10.1002/rcs.70040. In the video, we summarise the background of the work and the operative modes used in the evaluation. Then, we show representative videos for the user studies on expert surgeon usability and novice user learning curve followed by the evaluation of autonomous positioning.

The varying quality of the hand‐painted circles can affect the positioning accuracy. To suppress this influence and prepare a shared criterion, the subjects were informed of the above definition beforehand and instructed to position the tip to the centre.

The unit was converted to μm from pixel using the diameter of a 25G needle as reference in the microscopic view, and the following results are represented in μm.

##### 2.3.4.2 Workload (NASA‐TLX)

The NASA Task Load Index (NASA‐TLX) [34] is frequently used as a tool for the subjective evaluation of workload. It provides an overall workload score based on a weighted average of ratings on six categories: Mental Demand, Physical Demand, Temporal Demand, Performance, Effort, and Frustration.

The relative weights between categories $\omega_{i}\;\left(i=1,\;2,\;\text{…},\;6\right)$ are obtained using a pairwise comparison sheet after the first trial. After each trial, subjects rate the six workload categories and give $v_{i}\in \left[0,20\right]\;\left(i=1,\;2,\;\text{…},\;6\right)$. In addition, they can add open comments at the end of the sheet. Using the weight, $\omega_{i}$, and the score, $v_{i}$, the weighted workload (WWL) is calculated as follows

(5) $$WWL=\frac{\sum_{i=1}^{6}\omega_{i}v_{i}}{\sum_{i=1}^{6}\omega_{i}}=\frac{\sum_{i=1}^{6}\omega_{i}v_{i}}{15}.$$

The maximum value of the WWL is 20, and a small WWL means a small workload.

##### 2.3.4.3 Force Applied on the Fundus

**FIGURE 7:**
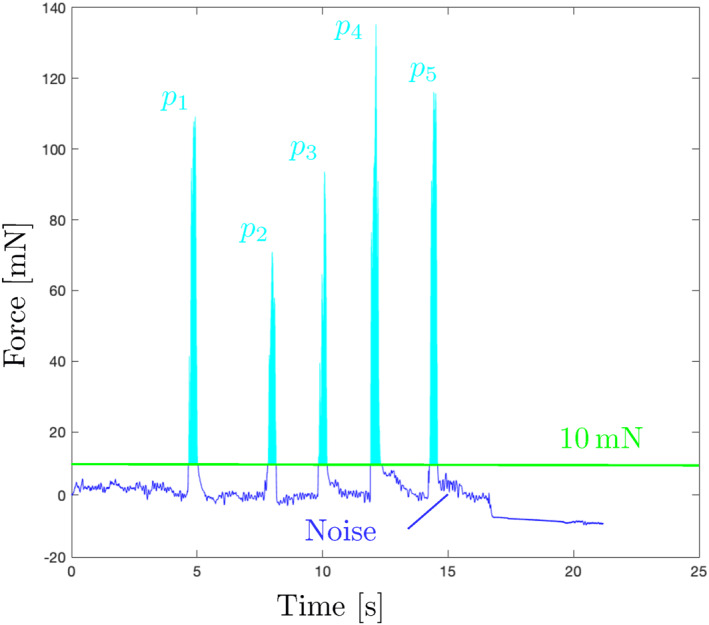
An example of the waveform of the force applied on the fundus and definition of the average force. The total area and the total time were used to calculate the average force.

The force applied on the fundus was measured using the BionicEyE with a quartz crystal resonator (QCR) [28] shown in Figure 5. The sensor enabled us to discuss the damage on the fundus in the depth direction on behalf of 3D measurement tools, such as an iOCT. The force resolution of the sensor is 0.22mN [This is the resolution under ideal conditions. In the user studies, additional sources of noise, such as the rotation of the Bionic‐EyE, are expected.]. As a metric for safety, we used the average force applied on the eye fundus. To attenuate the low‐amplitude noise, the average force was calculated above a 10mN threshold[The complete noise characterisation of the sensor and Bionic‐EyE could allow for a more robust filtering strategy, but that is out of the scope of this work.]. An example of this can be visualised in Figure 7.

##### 2.3.4.4 Task Duration

In the robot‐assisted surgical setups, the task duration was defined as the time‐span between the first time the clutch was pressed to the last time the clutch was released. In the case of the manual setup, the task duration was calculated from the recorded videos.

### 2.4 Design of Experiment E1: Evaluation of Autonomous Positioning

In the previous work [26], we proposed an autonomous positioning strategy that uses the shadow as a cue to estimate the proximity between the instrument's tip and the fundus. The visibility of the shadow is guaranteed by the constraint $C_{1}$ described in Section 2.2.4. The purpose of this experiment was to evaluate our autonomous positioning method against the ones involving an operator.

The following sections first describe our autonomous positioning method and, then, the improvements from the previous work in image processing.

#### 2.4.1 Four‐Step Strategy of Shadow‐Based Autonomous Positioning

**FIGURE 8:**
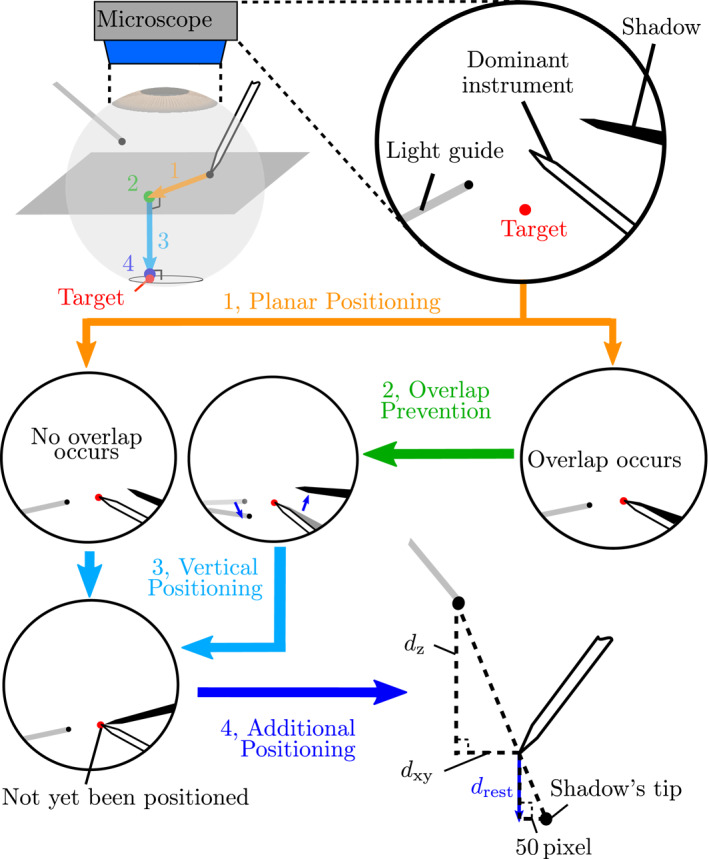
The four‐step strategy of the shadow‐based autonomous positioning. If the overlap of the dominant instrument and its shadow occurs after the first step, the controller tries to reduce the overlap in the second step. In the last step, the distance between the instrument's tip and the fundus after the third step is calculated using the geometrical relationship of the instrument and its shadow, and the instrument is moved downward to complete positioning.

Our strategy is divided into the following four steps as shown in Figure 8. The controller autonomously switches between steps based on the information obtained from microscopic images.

1. *Planar Positioning*: Firstly, the instrument's tip is moved parallel to the image plane to a safe point above the target point. This step converges when the distance between the instrument's tip and the target, $d_{\text{planar}}$, is less or equal to 20pixels (approximately 50μm).
2. *Overlap Positioning*: This step ensures that the instrument's tip and its shadow do not overlap. This step runs until the distance between the shadow's tip and the instrument's shaft, $d_{\text{shaft}}$, gets equal to or larger than 100pixels (approximately 240μm). In our experiments, the overlap was always avoidable, but if not the automatic controller stops here.
3. *Vertical Positioning*: In this step, the instrument is moved vertically to the target on the fundus of the eye. This step converges when the distance between the surgical instrument's tip and the shadow's tip, $d_{\text{tip}}$, is less or equal to 50pixels (approximately 120μm).
4. *Additional Positioning*: The instrument's tip does not reach the fundus after the vertical positioning step. Therefore, the controller needs to move the instrument further. The remaining distance after the vertical positioning step, $d_{\text{rest}}$, can be calculated using the geometrical relationship, illustrated in Figure 8, as follows

(6) $$d_{\text{rest}}\;\left[\mu m\right]=50\;\left[px\right]\times \frac{d_{z}\;\left[\mu m\right]}{d_{xy}\;\left[\mu m\right]}\times \frac{1000}{s\;\left[px/mm\right]},$$

where $d_{xy}$ and $d_{z}$ are the xy and z components of the relative position of the light guide's tip and the instrument's tip, respectively. The scaling factor, s=417[px/mm], was obtained using the diameter of the 25G surgical needle. Moreover, to ensure positioning, the controller moves the instrument

(7) $$d_{\text{add}}=d_{\text{rest}}+130\;\left[\mu m\right].$$

The value of 130[μm] was determined based on the variation of $d_{\text{rest}}$ obtained in a preliminary evaluation; the mean value was 50[μm] with the standard deviation of 80[μm].

The mathematical details of the control laws of each step are described in Section V‐VII of our previous work [26], respectively [The third and fourth steps share the same control law.].

##### 2.4.1.1 Improvements in Image Processing

The distances, $d_{\text{planar}}$, $d_{\text{shaft}}$, and $d_{\text{tip}}$, are obtained from microscopic images and used to switch between the steps. In our previous work [26], the accuracy and precision were affected by the relatively low resolution of microscopic images $\left(s_{\text{old}}=14\;\text{pixel}/mm\right)$ and low refresh rate (11Hz). Therefore, these points were addressed by improvements on the setup and in the network, resulting in an improvement of about 30 times for the scale factor, s=417pixel/mm, and around six times improvement in refresh rate, 60Hz.

##### 2.4.1.2 Flow of Image Processing

**FIGURE 9:**
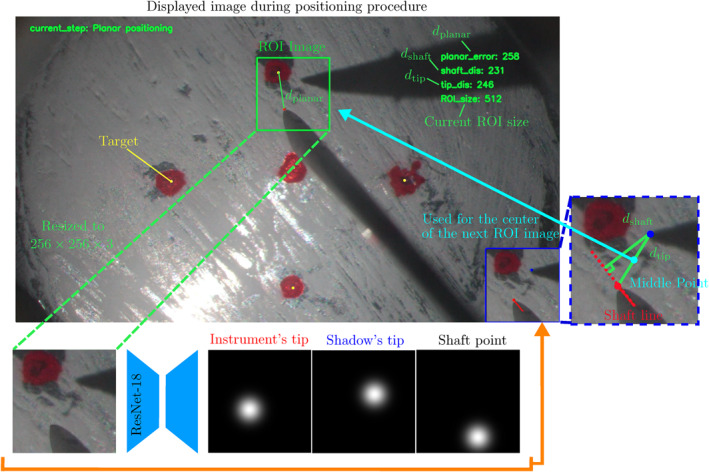
The image processing flow of calculating $d_{\text{planar}}$, $d_{\text{shaft}}$, and $d_{\text{tip}}$. The ROIs are extracted from the captured 4K microscopic images (3840×2160×3). Then, three representative points needed to calculate $d_{\text{planar}}$, $d_{\text{shaft}}$, and $d_{\text{tip}}$ are predicted using a UNet‐based model with the outputs of three confidence maps.

To calculate $d_{\text{planar}}$, $d_{\text{shaft}}$, and $d_{\text{tip}}$ from a microscopic view, the shaft line of the instrument and the tip positions of both instrument and shadow need to be predicted. For this, a UNet‐based image processing was implemented as shown in Figure 9.

Firstly, microscopic images (3840×2160×3) are obtained using a 4K camera (STC‐HD853HDMI, Omron‐Sentech, Japan). To keep the resolution of the image and achieve a short processing time, a region of interest (ROI) is set. Three ROI sizes are used, namely 768×768×3, 512×512×3, and 256×256×3. The active size for the ROI is selected based on the distance between the tips of the previous image so that the smaller size is used as the tips get closer. The centre of the ROI corresponds to the middle point of the tip points of the instrument and shadow in the previous image. For the first captured image, the ROI size is set to 768×768×3, and the centre is set arbitrarily.

Then, an extracted ROI image is resized to 256×256×3 [When the size of the active ROI is 256×256×3, the extracted image is not resized, that is, kept in 4K quality.] and input to the prediction model. The outputs of the network are three confidence maps that predict the positions of the instrument's tip, the shadow's tip, and a point in the instrument's shaft. The point in the instrument's shaft is used to estimate the shaft line, or the centre line of the instrument, by connecting it with the predicted instrument's tip. For this, a point in the shaft line was randomly chosen as the ground truth in the training data. The distances, $d_{\text{shaft}}$, and $d_{\text{tip}}$, are calculated as the distances from the predicted shadow's tip to the predicted instrument's shaft and tip, respectively.

The distance $d_{\text{planar}}$ is calculated as the distance between the predicted instrument's tip and the target point. The positions of the targets are detected using a computer vision library, OpenCV [https://opencv.org/], as red areas in HSV space (H: 0−30,150−179 [In OpenCV (https://opencv.org/), the hue values are normalised into 0–179°, and red corresponds to these two continuous areas.], S: 64−255, V: 70−255) before starting autonomous positioning and fixed during the whole process since the eyeball does not rotate much because of the fixed insertion points.

##### 2.4.1.3 Training of Network Model

**FIGURE 10:**
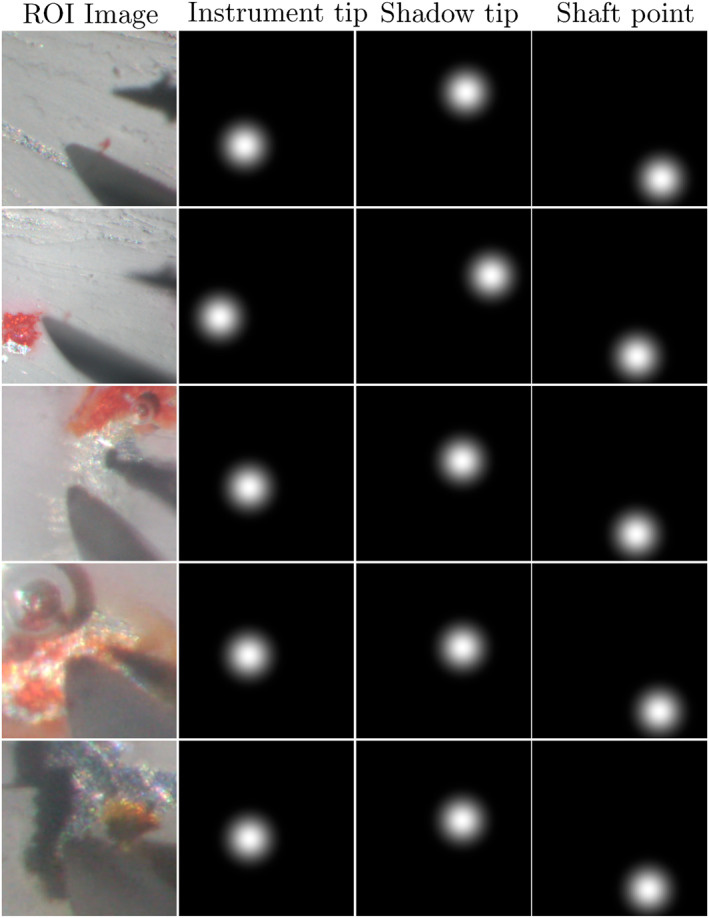
Samples of the ground truth. Three confidence maps are prepared for one microscopic image. These confidence maps represent, from left to right, the positions of the instrument's tip, the shadow's tip, and a point in the instrument's shaft used to predict the shaft line, respectively. During the experiment, bubbles were generated because of electrolysis, and the paint was peeled to expose the aluminium sheet. These images are also included in the training data.

The model was implemented based on a PyTorch library [35], using a ResNet‐18 pre‐trained on ImageNet as encoder. To prepare learning data, we collected 600 microscopic images and created ground truth images as shown in Figure 10 [We are going to extend the image segmentation network model to real intraocular images in future work. Please note that our current focus is the evaluation and comparison of our robot control strategy itself.]. Moreover, common augmentation strategies were used: blur, additive noise, image compression, and random gamma [36]. Considering all augmentations, we had 2500 images for training, 250 images for validation, and 250 images for testing.

**FIGURE 11:**
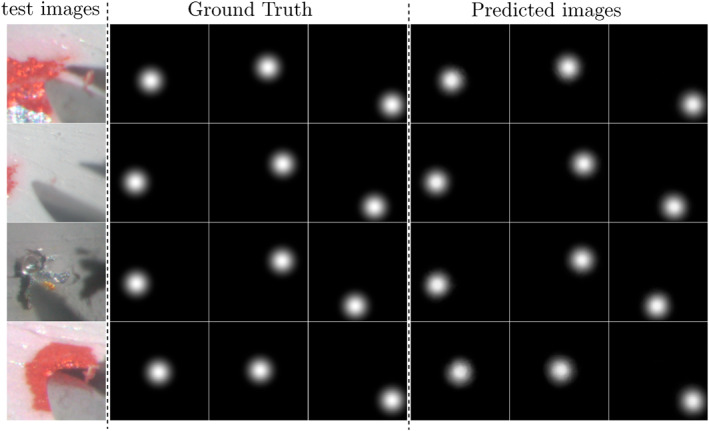
Predicted images of the test images. The images represent, from left to right, a test image, its corresponding three ground truth confidence maps, and the predicted confidence maps, respectively. The model was evaluated using Mean Squared Error (MSE) loss, and the MSE loss on the test set was $2.127\times 10^{-4}$.

The network is trained using Mean Squared Error (MSE) loss, Adam optimiser with a learning rate of 0.0001, and batch size of 5. The network was trained on a single NVIDIA GeForce RTX 3090 GPU. The MSE loss on the test set was $2.127\times 10^{-4}$. The performance of our algorithm depends on the accuracy of the model since the post process is a sequence of calculations that uses the estimated points. Therefore, this test results verified our image processing. Figure 11 shows representative examples of the prediction.

## 3 Results

This section describes the results of the two user studies and one experiment explained in Section 2.3 and 2.4, respectively. For the two user studies, the results of statistical analysis are also provided.

### 3.1 Results of the User Study U1

**FIGURE 12:**
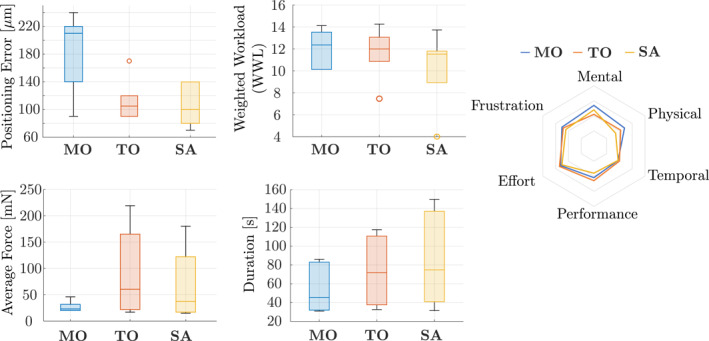
The results of the comparison of the three setups by expert surgeons.

Figure 12 shows the surgeons' performances with each setup. One‐sample Kolmogorov‐Smirnov tests [37] (https://www.mathworks.com/help/stats/kstest.html?s) indicated that no sample groups in any metric were normally distributed. This result justifies our choice of using non‐parametric tests.

The Kruskal‐Wallis test [38] (https://www.mathworks.com/help/stats/kruskalwallis.html?s) was first conducted for all metrics, but it indicated no significant differences among the three setups. Then, to compare **MO** against the robot‐assisted setups, **SA** and **TO**, we ran a post‐hoc Dunnett's test [39] (https://www.mathworks.com/help/stats/multcompare.html?s) with **MO** being the control group. A significant difference (p=0.0383<0.05) in positioning error was found between **MO** and **SA**.

Table 1 shows median values of the five trials in each setup, and Table 2 summarises each surgeon's relative weights between six categories of the NASA‐TLX, $\omega_{i}$ in (5), used to calculate WWL. In addition, Table 3 summarises the comments made by the surgeons.

**TABLE 1:** The median values of the five trials in each setup.

Surgeon #	Error $\left[\times 10^{2}\;\mu m\right]$			Workload			Average force [mN]			Duration [s]		
	MO	TO	SA	MO	TO	SA	MO	TO	SA	MO	TO	SA
1	2.0	1.7	1.4	13.5	10.9	**4.0**	**46**	165	122	32	40	**31**
2	2.2	1.2	1.4	13.0	12.1	**13.7**	27	**219**	**180**	34	**33**	42
3	2.4	0.9	0.9	11.7	13.1	11.4	**20**	37	17	**86**	**118**	108
4	1.4	0.9	0.8	**10.1**	**7.5**	8.9	**20**	22	23	83	111	137
5	2.2	1.1	0.7	**14.1**	**14.3**	11.7	a	84	52	**31**	38	41
6	0.9	1.0	1.1	**10.1**	11.9	11.8	23	**17**	**15**	57	103	**150**
Median	2.0	1.0	1.0	12.4	12.0	11.5	25	61	38	46	72	75

^a^ The contact detection system did not correctly work.

**TABLE 2:** Each surgeon's relative weights between six categories of the NASA‐TLX.

Surgeon #	1	2	3	4	5	6
Mental demand	4	2	1	3	3	3
Physical demand	2	0	2	5	2	1
Temporal demand	5	5	3	0	0	0
Performance	0	1	4	4	2	3
Effort	3	3	2	1	3	3
Frustration	1	4	3	2	5	5

**TABLE 3:** Comments made by the expert surgeons during the experiment.

Manual setup (MO)
There was more tension than in the robot‐assisted surgical setup.
The instruments interfered with the eyelid of the simulator.
The eye model suddenly rotated.
It was more difficult than usual when going to the left side in the microscopic view (the surgical needle was inserted from the right side).
**Robot‐assisted surgical setup (TO and SA)**
It was not easy to pinpoint the targets and touch.
The second trial was more difficult.
I Got used to the trial after the eighth time.
The foot switch should be more to the right.
In the **TO**, the light guide hardly needed to be operated.
The tip touched the fundus when returning the surgical needle after the positioning (the tip did not retract up).
I Need to get used to operating the robots more.
The operation was difficult because the direction of moving the instruments was different from the actual surgery.
The input interface was stuck at the limit of its range of motion.
It was difficult to move the instrument precisely during the last positioning movement.
**Overall**
2‐D images were difficult to estimate the distance to the fundus.
The target point $p_{5}$ seemed to be in the back.
Positioning the tip to the target points $p_{1}$ and $p_{5}$ was difficult.
Debris and bubbles in the fundus made it difficult to see the tip.

### 3.2 Results of the User Study U2

**FIGURE 13:**
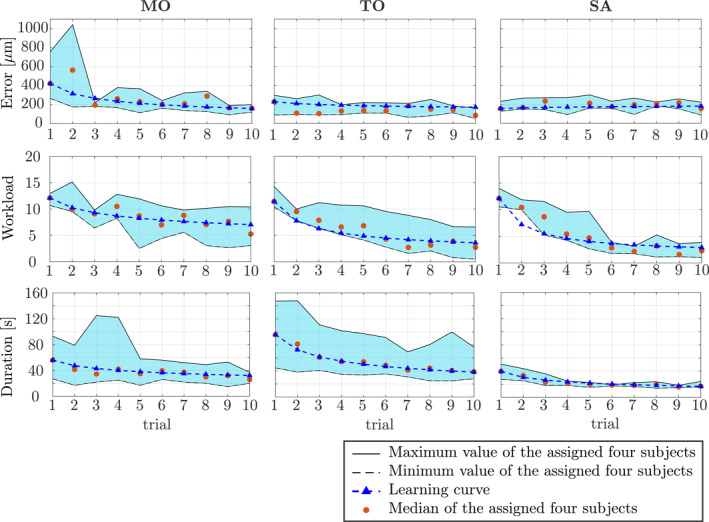
The learning curves of the positioning accuracy, workload, and the task duration.

The Wilcoxon signed‐rank test [40] (https://www.mathworks.com/help/stats/signrank.html) was first conducted to evaluate if learning was effective in each metric by comparing the first and last trials, combining all setups. The results indicated that there were significant differences in the positioning accuracy (p=0.0068<0.05), the workload (p=0.0005<0.05), and the task duration (p=0.0015<0.05). The learning curves of these metrics are shown in Figure 13. There was no significant difference in the average force.

Then, in each metric shown in Figure 13, the Kruskal‐Wallis test was conducted for the learning rate, the learning plateau, and AULC. No significant differences were indicated for the learning plateau in any metrics.

The learning rate had significant differences in the positioning error (p=0.0435<0.05) and the workload (p=0.0435<0.05). In both metrics, the post‐hoc Tukey‐Kramer test [41] (https://www.mathworks.com/help/stats/multcompare.html?s) indicated that there were significant differences between **MO** and **SA**: positioning error (p=0.0378<0.05) and workload (p=0.0378<0.05).

For AULC, there was a significant difference in the task duration (p=0.0435<0.05). The post‐hoc Tukey‐Kramer test resulted in a significant difference between **TO** and **SA** (p=0.0378<0.05).

### 3.3 Results of the Experiment E1

The system conducted the same positioning task as the user studies and positioned the needle's tip to the five target points in numerically crescent order. The trial was repeated until we obtained five successful executions. The controller fails, for instance, when there were difficulties with the autonomous image processing, such as dirt on the tip of the instrument (e.g. bubbles) and damages to the fundus of the eye model (see Figure 10). These factors are related to the nature of the repetitive use of the same experimental setup for a large number of trials, and not problems with the control strategy.

In total, seven trials were conducted. Table 4 shows the results of autonomous positioning to each point in the seven trials. The third and fifth had failures and were excluded. The star ⋆ means that the tip did not reach the fundus though the controller conducted the final additional positioning step. The dagger † means that the tip was positioned correctly in the final additional step. Table 5 shows the median of the evaluation metrics of the five successful trials.

**TABLE 4:** Results of autonomous positioning.

	$p_{1}$	$p_{2}$	$p_{3}$	$p_{4}$	$p_{5}$
Trial 1	b	a	b	b	b
Trial 2	b	b	b	b	a
Trial 3	b	a	c	c	c
Trial 4	b	b	b	b	b
Trial 5	b	c	c	c	c
Trial 6	a	a	a	b	a
Trial 7	b	b	b	a	a

^a^ Not positioned.

^b^ Positioned.

^c^ Force‐quit.

## 4 Discussion

Based on the results summarised in Section 3, we discuss the performance of the proposed one‐hand teleoperative control system and the fully autonomous positioning method in this section. Finally, we describe the conclusion of this work and the direction towards future work.

### 4.1 Discussion of the User Study U1

In the user study **U1**, the different control strategies were evaluated among expert surgeons. Since a significant difference was found between **MO** and **SA**, it can be said that **SA** can improve the positioning accuracy of expert surgeons.

From the comments summarised in Table 3, we can see that the surgeons did not fully get used to the operation of the interfaces and felt more tension in the robot‐assisted setups. This might have lead to the result that some surgeons felt more workload in the robot‐assisted setups as shown in Table 1.

The surgeons also mentioned that the light guide did not need to be actively operated in **TO** since the task did not require continuous manoeuvres of the light guide. This indicates that the task was too simple and that autonomous lighting did not contribute to efficiency. This might be the reason that **SA** did not shorten the task duration.

**FIGURE 14:**
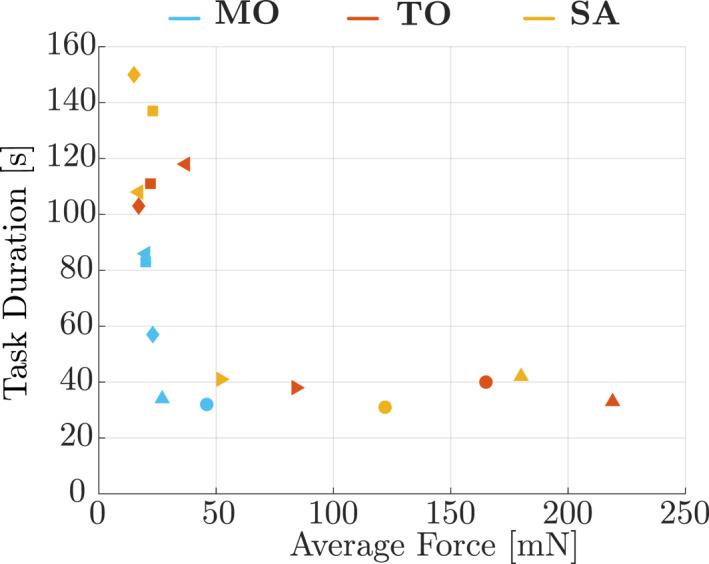
Each surgeon's performance plotted with average force and task duration on the axes. Each symbol represents a subject, and the colours correspond to each setup.

Based on the results of the task duration and the average force in Table 1, the surgeons can be divided into two groups; the surgeons who emphasised task duration (Surgeons 1, 2, and 5) and those who prioritised average force (Surgeons 3, 4, and 6). Figure 14 shows the results plotted with average force and task duration. It turned out that these metrics are incompatible with each other even in **MO**. When the robot‐assisted setups were used, this tendency was more pronounced. The surgeons who focused on the task duration in **MO** also showed the same behaviour in **TO** and **SA**, and vice versa. We could not find a specific factor that separates the two groups.

### 4.2 Discussion of the User Study U2

The user study **U2** evaluated the learning curves of novices. From the significant differences in the learning rate of the positioning error and the workload between **MO** and **SA**, we conclude that.

- the novices can achieve a consistent accuracy more quickly when using **SA**, and
- the novice's workload reduces more quickly when using **SA.**

A smaller value of AULC means that the task is easier for the subjects to learn. Therefore, from the significant difference in the AULC of the task duration between **TO** and **SA**, it can be said that **SA** can make it easier for the novices to learn to conduct the task quickly.

### 4.3 Discussion of the Experiment E1

We conducted the experiment **E1** to evaluate our fully autonomous positioning method against the ones involving an operator. To achieve equivalent performance to the surgeons.

We can say from Table 5 that we need to improve the average force and the task duration; the median of the surgeons are $2.0\times 10^{2}\;\mu m$ for positioning accuracy, 25mN for average force, and 46s for task duration. Please note that we can not compare the results with surgeon's results directly due to the difference of the experimental protocols. The average force was affected by the error included in $d_{\text{rest}}$ (see (6)). The error is mainly derived from the relative distances of the instrument tips, $d_{xy}$ and $d_{z}$. The main cause can be a calibration error in the relative positions of the robots.

**TABLE 5:** The median of the five successful trials.

Metric	Error $\left[\times 10^{2}\;\mu m\right]$	Average force [mN]	Duration [s]
Median	0.6	49	257

The long task duration was caused by the time for the planar positioning step to converge, which is affected by the refresh rate of the microscope. Because of this, the robot must move relatively slow at about 0.2mm/s during the planar positioning, resulting in 58% of the time being spent for the planar positioning.

These results add to our current knowledge that proper calibration affects the results of model‐based approaches such as this one. In addition, high‐accuracy offline calibration has been shown insufficient. In future work, we will explore an online calibration strategy [42], which we believe is the single most important source of further improvement.

## 5 Conclusion

In this work, we achieved a novel one‐hand teleoperative control system and conducted experiments to compare a manual surgery, a regular two‐hand teleoperation, and the proposed one‐hand teleoperation. Through the user studies, we showed that.

- the proposed one‐hand teleoperation could improve the positioning accuracy of expert surgeons,
- enabled novices to achieve a consistent accuracy more quickly than the manual setup,
- decreased the novice's workload more quickly than the manual setup, and
- made it easier than the regular teleoperation for novices to learn to conduct the task quickly.

Moreover, we evaluated our autonomous positioning strategy using the same positioning task. With improvements on the setup and in the image‐processing network, our proposed strategy successfully positioned the instrument's tip to the fundus of the eye model with an equivalent accuracy to the surgeons.

These results also gave us the followings as open issues, and they will be the targets of our future work:

- the benefit of task autonomy in a more complex surgical scenario, such as cannulation or peeling,
- the impact of more intuitive user interface and feedback modality on experimental results, and
- the impact of an online calibration strategy on the accuracy and safety of autonomous *positioning.*

## Author Contributions

**Murilo M. Marinho:** conceptualisation (lead), methodology (lead), formal analysis (supporting), software (supporting), supervision (supporting), writing–review & editing. **Yuki Koyama:** data curation, formal analysis (lead), investigation (lead), software (lead), writing–original draft preparation. **Yuta Taniguchi:** resources, investigation (supporting). **Toshiro Yamanaka:** investigation (supporting). **Fumihito Arai:** investigation (supporting), supervision (supporting), funding acquisition (supporting). **Seiji Omata:** investigation (supporting). **Koichiro Sugimoto:** investigation (supporting). **Takashi Ueta:** investigation (supporting). **Kiyohito Totsuka:** investigation (supporting). **Tomoyasu Shiraya:** investigation (supporting). **Fumiyuki Araki:** investigation (supporting). **Muneyuki Takao:** investigation (supporting). **Mamoru Mitsuishi:** supervision (supporting), funding acquisition (supporting). **Kanako Harada:** conceptualisation (supporting), supervision (lead), funding acquisition (lead).

## Ethics Statement

No patient data was used. All participants gave written consent after receiving an explanation regarding the experiment. The experimental protocol was approved by the Research Ethics Committee of the Faculty of Medicine of the University of Tokyo (Protocol number: 2022174NI, Date of Approval: October 3rd, 2022).

## Conflicts of Interest

The authors declare no conflicts of interest.

## Data Availability

Data not made available to preserve the identity of the participants.
